# Vocal pain expression augmentation for a robopatient

**DOI:** 10.3389/frobt.2023.1122914

**Published:** 2023-09-13

**Authors:** Namnueng Protpagorn, Thilina Dulantha Lalitharatne, Leone Costi, Fumiya Iida

**Affiliations:** ^1^ Bio Inspired Robotics Laboratory, Department of Engineering, University of Cambridge, Cambridge, United Kingdom; ^2^ Dyson School of Design Engineering, Imperial College London, London, United Kingdom

**Keywords:** robotic patients, robot-assisted training, audio augmentation, human–robot interaction, palpation, medical training simulators

## Abstract

Abdominal palpation is one of the basic but important physical examination methods used by physicians. Visual, auditory, and haptic feedback from the patients are known to be the main sources of feedback they use in the diagnosis. However, learning to interpret this feedback and making accurate diagnosis require several years of training. Many abdominal palpation training simulators have been proposed to date, but very limited attempts have been reported in integrating vocal pain expressions into physical abdominal palpation simulators. Here, we present a vocal pain expression augmentation for a robopatient. The proposed robopatient is capable of providing real-time facial and vocal pain expressions based on the exerted palpation force and position on the abdominal phantom of the robopatient. A pilot study is conducted to test the proposed system, and we show the potential of integrating vocal pain expressions to the robopatient. The platform has also been tested by two clinical experts with prior experience in abdominal palpation. Their evaluations on functionality and suggestions for improvements are presented. We highlight the advantages of the proposed robopatient with real-time vocal and facial pain expressions as a controllable simulator platform for abdominal palpation training studies. Finally, we discuss the limitations of the proposed approach and suggest several future directions for improvements.

## 1 Introduction

Medical errors not only lead to increased mortality rates in patients (Donaldson et al., 2000) but also contribute to huge financial loss (Elliott et al., 2021) to countries’ economies. Improvements in training of medical professionals by diversifying training methods and increasing the training frequency (Davis et al., 1995; Likic and Maxwell, 2009) are important to minimise these errors and/or accidents. Medical training is an essential but time-consuming process that medical students and practitioners need to go through to improve their skills. Abdominal palpation is one of the primary examination methods used by physicians to examine the abdomen of patients, and it often takes years of training (Danielson et al., 2019) to become skilful in palpation. Palpation is a multi-sensory motor coordination task (Lalitharatne et al., 2020) that involves a physician interpreting haptic, visual, and auditory feedback from the patient under diagnosis and controlling his/her palpation behaviour accordingly to accurately perform the diagnosis. Moreover, the expression of pain, both visually and verbally, is often used during the procedure to correctly identify the correct location of the tender tissue and the organ to which such tissue belongs (Rastogi et al., 2018).

Given the risks associated with training on actual patients, simulation-based education (SBE) has been introduced for training medical students. SBE enables a safe and controlled environment for medical students to practice their physical exami nation skills including abdominal palpation. SBE offers tailored training experiences for students whereby mistakes and errors can be made before practicing on real patients that helps to reduce complications and liabilities. Among different methods of SBE, standardised patients (SPs), who are professionally trained actors acting as patients, have been the highest fidelity simulators that could be found in the medical field for decades (Costanza et al., 1999). The SPs are capable of replicating facial expressions, vocal or audio expressions, both in the form of meaningful sentences or unstructured grunts, and variations of muscle stiffness of patients during palpation training. Training with SPs has been reported as effective (Walker et al., 1990), but it is time consuming given the lengthy training time needed to become SPs and their skill maintenance. It also can lead to several encounter-based biases depending on the specific interaction between a given student and a particular SP (Fluet et al., 2022). Alternatively, virtual and/or physical training simulators have been proposed to address the issues related to SPs. Medical training simulators like the robopatient (Lalitharatne et al., 2021; Lalitharatne et al., 2022; Tan et al., 2022) enable medical students to practice hands-on medical skills on demand, as opposed to on limited resources of professional actors or real patients (Walker et al., 1990). These technologies have been proven to lead to better development of the required skills by medical students (Schubart et al., 2012).

Computer-based palpation training simulators that utilise visual feedback include visualising the colours and texture of tumours (Shafikov et al., 2020) or generating virtual patients (Kotranza and Lok, 2008; Wandner et al., 2010; Rivera-Gutierrez et al., 2012; Maicher et al., 2017; Daher et al., 2020). However, virtual simulators lack the physicality that has been identified as an important aspect of medical training (Chuah et al., 2013; Pan and Steed, 2016). On the other hand, physical simulators, such as manikins, often provide a haptic feedback. The feedback, in particular real-time feedback, is an essential tool for the trainees. Thus, with the aim to provide the trainees with this important tool, incorporation of sensors, actuators, and algorithms in high-fidelity simulators or manikins is essential. Sensors are needed to capture important data during training sessions, actuators will be used to generate realistic responses, and algorithms are necessary to analyse the data and deliver the feedback in real time. Haptic feedback setups for palpation application range from modelling tumours using granular jamming (Rørvik et al., 2021) to physical systems using interchangeable organs (Rethy et al., 2018). Some physical simulators also produce visual cues, such as the physical–virtual hybrid face rendering pain facial expressions (Lalitharatne et al., 2021). Also, a study to find the effect of the real-time auditory feedback during palpation of a simulated lumbar spine has been reported (Gugliotti et al., 2019). However, their simulator consisted of only auditory pain feedback without facial expressions.

In general, communicating pain through vocal expressions is not a trivial task. Making pain sounds or vocal expressions is the form of communication that exists pre-language and is used by animals as well as young children (Scarry, 1985). Variations in pain intensities and human backgrounds, such as gender and ethnicity, lead to variations in the pain sound expressions. In a study conducted by Raine et al. (2019), trained actors were asked to produce pain sounds corresponding to three levels of pain intensity. A group of listeners were asked to rate the pain intensity between 0 and 100 based on the sounds produced. The result has shown that the difference in voice features of the trained actors could lead to up to 76% variation across the pain levels and 54% within the pain levels. Additionally, the level of stimulated pain intensity encoded in the pain vocalisation non-linearly increases as the mean and range of the fundamental frequency, amplitude, and degree of periodicity increase. Similar research has been reported on infants. When infants experience a higher intensity of pain, they produce more irregular cries (Tiezzi et al., 2004; Facchini et al., 2005; Konstantinova et al., 2017) with higher amplitude (Fuller and Conner, 1995; Lehr et al., 2007; Maitre et al., 2017), larger fundamental frequency variation (Porter et al., 1986; Koutseff et al., 2018), smaller amplitude variation, and longer duration (Porter et al., 1986; Johnston and OʼShaugnessy, 1987). Higher pitch and louder pain cries are associated with a sense of urgency (Porter et al., 1986; Craig et al., 1988). Pain vocalisation is an important indicator for much medical attention, such as during child labour (Fuller et al., 1993). Humans vocalise different types of pain sounds in different contexts (for example, vocal pain expressions during abdominal palpation vs. when someone touches a hot object) which makes modelling the pain sounds or vocal pain expressions challenging (Lautenbacher et al., 2017; Helmer et al., 2020). To the best of our knowledge, studies of pain vocalisation in the abdominal palpation context are difficult to find and has not been applied to physical abdominal palpation simulators. Despite all these existing works, a physical abdominal palpation simulator that is capable of synthesising vocal and facial expressions has not been reported.

To fill these gaps, in this paper, we present a controllable and customisable physical robopatient with facial and vocal pain expressions for abdominal palpation training. The robopatient setup consists of a robotic face called MorphFace (Lalitharatne et al., 2021) and a force sensor platform. The proposed system is able to render real-time pain facial expressions and vocal pain expressions given the user palpation force and position on a silicone abdominal phantom. We proposed a simple yet effective threshold-based algorithm that was built upon our previous study (Protpagorn et al., 2022) for generating the vocal pain expressions based on the estimated pain intensity of the robopatient. Here, we develop a physical setup that allows participants to perform palpation, replicating a real examination experience instead of using an optical mouse as in our previous work. In order to test the feasibility and performance of the proposed robopatient with vocal and facial pain expressions, we conducted a pilot study with naive participants (*n* = 8) and clinical experts (*n* = 2) who had prior experience in palpation to compare this approach with a robopatient that consists of only facial pain expressions. In the user studies, participants are asked to estimate the maximum pain point/location by palpating the silicone phantom while observing facial pain expressions or facial and vocal pain expressions. Additionally, qualitative feedback on the functionality of the proposed platform and recommendations for improvements are recorded from the clinical experts.

The rest of the paper is organised as follows: Section 2 discusses the development of the proposed robopatient with vocal pain expressions that includes details of the hardware integration, palpation to pain intensity mapping process, and pain intensity to vocal pain expressions generations. Experiments and results are presented in Section 3. Finally, in Section 4, a discussion of limitations of the current study and possible future directions are presented.

## 2 Methods

**FIGURE 1:**
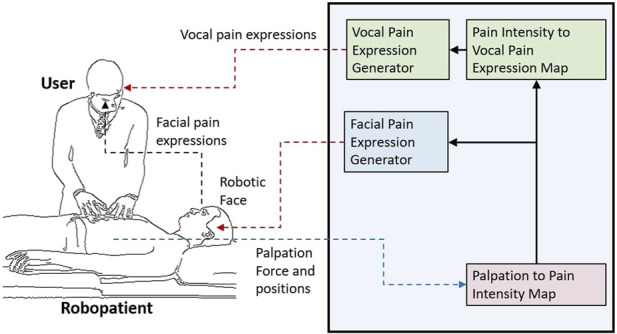
Overview of the proposed robopatient with vocal pain expressions. It consists of three stages: palpation to pain intensity mapping, pain intensity to vocal expression mapping and vocal expression generation, and facial pain expression generations. The interface takes the user palpation force and position as the input and generates pain facial and vocal expressions as outputs in real time.

**FIGURE 2:**
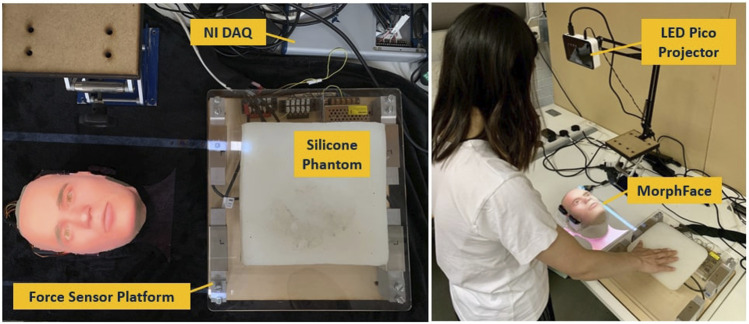
Developed robopatient with vocal pain expression. The MorphFace generates different facial expressions according to the facial pain expression generator. Audio pain expressions are vocalised according to the vocal pain expression generator and played using the laptop’s speakers.

The overview of the proposed robopatient with vocal pain expressions is depicted in Figure 1. It mainly consists of three stages: palpation to pain intensity mapping, pain intensity to vocal expression mapping and vocal expression generation, and facial pain expression generations. The developed robopatient is shown in Figure 2. It contains a force sensor platform, a silicone phantom that mimics the abdomen of a patient, and a robotic face called MorphFace (Lalitharatne et al., 2021). The silicone phantom is 20 × 20 × 5 *cm* ^3^ in dimension, and it is fabricated by silicone casting Ecoflex 00-20 (Smooth-On Inc., United States) in a 3D printed PLA mold. The monomer and the crosslinker of the silicone are mixed for 2 min, placed in a vacuum for 20 min, and then, cast and cured into the mold. The excess material has been removed using a scalpel.

### 2.1 Palpation to pain intensity mapping

In order to map the palpation behaviour with the pain intensity, we first acquire the user palpation data using a force sensor platform. This platform is built with four single-axis Tedea Huntleigh 1,040 (20 *Kg*) load cells attached to the corners of a square 290(*L*)*X*310(*W*)*X*5(*H*)*mm* acrylic board, as shown in Figure 2 (left). To acquire the signals from the load cells, a National Instrument DAQ (USB-6341) is used. These data are recorded using MATLAB 2020a (Mathwork Inc.) at a sampling rate of 250 Hz. Raw signals from the load cell were pre-processed with a moving average filter (window size: 20) and then used for palpation force and position calculation. We estimated the total palpation force (*F*_*tot*_) and position of the palpation along the x-direction (*x*) using the following equations:

$$F_{\mathrm{tot}}=\overset{4}{\underset{j=1}{\sum }}F_{j},$$ (1)

$$x=\left(F_{2}+F_{3}\right)\frac{d}{F_{\mathrm{tot}}},$$ (2)

where *F*_*j*_ is force readings of *j*th load cell placed at the four corners of the force sensor platform and *d* is the distance between two adjacent load cells.

To simulate the pain intensity on the robopatient abdomen caused by a painful edge, e.g., the liver, we generated a pain map using a radial basis interpolation using a Gaussian function. We assumed that the pain intensity is defined by the Gaussian function across the entire phantom surface *N*(*μ*, *σ* ^2^), where *μ* is the painful edge and *σ* is the standard deviation indicating the spread of the pain. Therefore, to estimate the pain intensity (*PI*) to be associated with facial expressions, the palpation position along the (*x*)-direction into the 1D Gaussian pain function is multiplied by *F*_*tot*_ as shown in the following equation:

$$\text{PI}=\frac{kF_{\mathrm{tot}}}{\sigma \sqrt{2\pi }}e^{-\frac{{\left(x-\mu \right)}^{2}}{2\sigma^{2}}},$$ (3)

where constant *k* is calibrated so that the pain intensity ranges between 0 and 100. *x* is the x-coordinate of the palpation, and *σ* is the standard deviation or spread of the pain function (here, we set *σ* = 25 empirically). Note that there are two factors determining the estimation of *PI*: the spatial distribution of pain and the applied force. This implies that applying high pressure or force can produce pain in any location in the abdomen. However, at the maximum pain point or edge, even a slight force would elicit intense pain or pain expressions.

Algorithm 1 Pain intensity to vocal pain expression mapping for the robopatient.

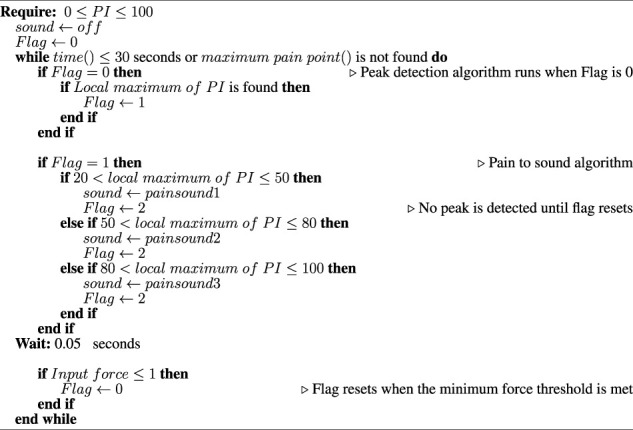

### 2.2 Pain intensity to vocal pain expression mapping and vocal pain expression generation

Mapping palpation force and position to vocal pain expressions is not a trivial task. Even though several studies on vocal pain sound when participants are exposed to external stimuli (Lautenbacher et al., 2017) have been reported, we could not find any study that attempted to map the vocal pain expressions using palpation. In our recent study (Protpagorn et al., 2022), we proposed a simple threshold-based algorithm to map the vocal pain expressions using palpation. Therefore, in the current study, we extend this threshold-based algorithm shown in 1 to generate the vocal pain sounds. In the previous algorithm (Protpagorn et al., 2022), only the mouse cursor input was considered, whereas the current algorithm takes into account both the palpation position (location) and the palpation force to calculate the pain intensity. This is advantageous as the new approach closely resembles a real palpation examination.

In the proposed threshold-based algorithm, three pain sounds are used to simulate the vocal pain expressions during the palpation. A prior study by Raine et al. (2019) showed that the frequency (pitch) and amplitude (volume) of pain vocalisation are increased as the pain intensity increases. Therefore, we map the *PI* to three discrete vocal pain expressions as follows: Pain sound 1 is derived from pain sound 2 with 0.7 × *frequency* and 0.5 × *amplitude*. Pain sound 3 is derived from pain sound 2 with 1.4 × *frequecncy* and 2 × *amplitude*. As for the pain sound 2, we use a male pain sound downloaded from a pain sound dataset (Protpagorn et al., 2022).

**FIGURE 3:**
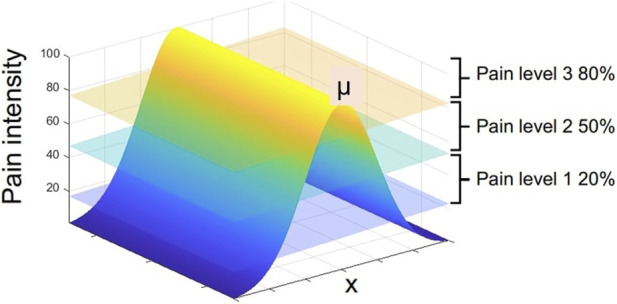
1D Gaussian pain intensity around the maximum pain point μ and the map of vocal pain expression generation according to Algorithm 1.

The algorithm generates three distinct pain level signals (i.e., pain sound 1, pain sound 2, and pain sound 3), and these are activated when a threshold is reached. A graphical representation of these thresholds is illustrated in Figure 3. We select three threshold values 20, 50, and 80 based on the user study by Raine et al. (2019) where 34 participants were asked to rate three levels of mild, moderate, and severe pain sounds. The average rating for mild, moderate, and severe pain sounds were 20, 50, and 80 (based on the Likert scale from 0 (no pain) to 100 (extreme pain)). To avoid playing multiple pain sounds at the same time, we use a time delay of 0.05*s* after each sound and make sure that no sound is played until the pain threshold is reset and the user palpate to achieve a local maximum again.

### 2.3 Facial pain expression generation

We use MorphFace Lalitharatne et al. (2021), a controllable 3D physical–virtual hybrid face, to represent pain expressions of patients. The pain facial expressions are realised using four facial action units (AUs), namely, AU4: brow lowerer, AU7: lip tightener, AU9: nose wrinkler, and AU10: upper lip raiser, which are used to synthesise the pain facial expressions. The intensity of each AU is controlled based on the value of *PI*, where 0 represents the no pain or neutral face and 100 represents the maximum painful face. More information about the MorphFace and its design and implementation can be found in the work of Lalitharatne et al. (2020), Lalitharatne et al. (2021), and Tan, (2021).

The focus of the study is to test the robopatient with vocal pain expressions rather than different identities of the face. While the MorphFace is capable of rendering faces of different sexes and ethnic background patients, we chose to use a white male face in our pilot study to simplify the experiments and minimise the influence of other variables, such as gender and ethnic identity of the face, on pain perception. The specific choice of a white male face may have limitations in terms of potential bias or lack of generalisability to other populations. In our future studies, we plan to investigate the impact of using faces from different ethnic backgrounds together with pain vocal expressions on pain perception.

## 3 Experiments and results

To test the system, we conducted a pilot user study with *n* = 8 naive participants and *n* = 2 clinical experts. The experiment consisted of two main tasks. In one task, we asked the participants (in the case of the experiment with naive participants; one female and seven males, aged between 21 and 35 (25 ± 6) years, undergraduate and postgraduate engineering students from the University of Cambridge who had no visual impairment or hearing difficulties) to estimate the location of a maximum pain point in the silicone phantom (which resembles an abdomen of a patient with painful liver edge) by palpating the silicone phantom while viewing the facial expressions displayed by the MorphFace. During the second task, we asked the same participants to repeat the experiment but instead of only facial pain expressions, they were presented with both the facial and vocal pain expressions.

At the beginning of the experiment, detailed oral instructions of the tasks were given and the participants signed a consent form that indicates the purpose of the experiment, what the experiment involves and how the data were handled. The experiment started with two calibration trials in which the participants were asked to familiarise themselves with the sensory cues from the robopatient. The participants palpated the silicon phantom in the centre, which is the location of the maximum pain point, to familiarise themselves with the facial expression feedback for 30 s. In the next calibration trial, the participants were asked to listen to three pain sounds corresponding to three levels of pain while palpating the silicone phantom for 30 s to recognise the vocal pain expressions.

After the calibration trials, the participants were asked to perform one of the experiment tasks (i.e., with only facial pain expressions or facial and vocal pain expressions) followed by the remaining task. We counterbalanced the order (i.e., pseudo-randomly varied the order (face only first vs. face + vocal first) of the experiments) of two tasks across participants to control for potential learning and order effects. We instructed the participants to palpate the silicon phantom in the x-direction starting from the right, as shown in Figure 2, and simultaneously analyse the facial expression or the facial and vocal pain expression to determine the maximum pain location. Even though there is no upper limit to how long a palpation examination can last in the actual patient examination, in our experiments, we set a limit to the exploration time. In the current study, we asked the participants to explore only in the x-direction, whereas in our previous palpation experiments (Lalitharatne et al., 2022), the participants have been asked to explore the phantom by palpating X–Y directions which was more demanding. However, even with X–Y exploration tasks, average time taken to find the maximum pain point was approximately 42–43s. Therefore, to avoid the participant getting fatigued by prolonged palpation and based on our preliminary studies, we set the limit of the exploration time to 30 s.

The trial and the 30 s timer started when a beep noise was made. The participants were asked to estimate the maximum pain point within 30 s. The final palpation location on the silicone phantom that the participants palpate before the trial is over was considered the estimated maximum pain point. If the maximum pain point/location is found before the 30 s timer is over, participants were asked to stop palpating and wait until the timer is over. If participants take longer than 30 s to find the maximum pain point, the current trial was automatically ended and the next trial was start after the beep. The maximum pain point *μ* was changed randomly between (100, 115, 130, 145, 160, 175 *mm*) in each trial. Each experiment task consisted of 10 trials resulting in 20 trials per participant. Raw and processed data (total palpation force and palpation position) were recorded during all trials. At the end of the experiment, qualitative feedback (regarding the participants’ strategy for finding the maximum pain point and the ease of making decisions with different feedback or combinations of feedback) was collected verbally and recorded by the experimenter.

The same procedure was followed during the experiments with two clinical experts: (1) an MD in medicine, a clinical fellow in critical care, with 10 years of experience, aged 36 years, female, right handed; (2) an MD-PhD candidate with 2 years of experience, aged 24 years, male, left handed. Immediately after the experiments, two clinical experts answered a 5-point Likert scale questionnaire, which consisted of the following questions: *(1) Were the vocal pain expressions realistic? (2) Were the vocal pain expressions clearly distinguishable*? *(3) Were the vocal pain expressions helpful in identifying the maximum pain point? (4) Is the simulator platform with vocal pain expressions useful or potentially useful in palpation medical training?* In addition, they also answered two open-ended questions. *(5) What needs to be improved in terms of vocal pain expressions? (6) What features would you like to see in a medical simulator with vocal pain expressions? *The experiment protocol was approved by the ethics committee of Department of Engineering, University of Cambridge, United Kingdom (Vocal Pain Expression Augmentation for a robopatient (low risk): 25/05/2022). All experiment protocols were performed in accordance with the relevant guidelines and regulations.

We analysed the recorded data using MATLAB 2020a (Mathworks Inc.). We filtered the palpation force data using a low-pass IIR filter with order 3 and a pass-band frequency of 10*Hz*. To remove the transient at the beginning of the trial, we discarded the first 100 data points. Then, we estimated the palpation peaks using the findpeak function in MATLAB with parameters set to 1, 2, and 100 for minimum peak prominence, minimum peak height, and minimum peak distance, respectively. Since resulting data consist of multiple peaks at each palpation, a custom algorithm is implemented to choose only the maximum peak within each palpation that registered a force greater than average palpation force of the particular trial. Given the final detected peak for each palpation, we used the corresponding index of the peak to find the respective time stamps in seconds and palpation positions. The participants could estimate the location of the maximum pain point in almost all trials within 30*s*. Four trials resulted in participants not being able to confirm the maximum pain point within this time limit. These trials were still included in the analysis, with the location of the maximum pain point and trial completion time estimated based on the last palpation peak and corresponding time stamp.

**FIGURE 4:**
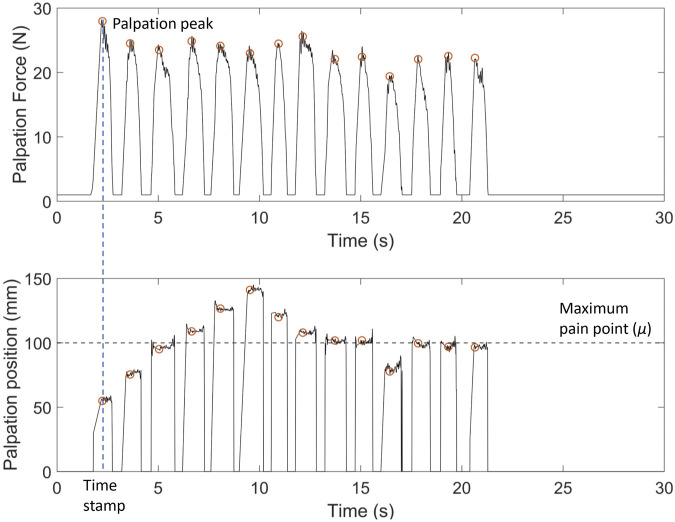
Example of palpation force (*N*) and palpation position (X-position) (*mm*) against time for one naive participant with facial expression-only feedback. Palpation point is the local maximum of the palpation force for each press. X-position of the palpation point is extrapolated from the time stamp. Here, *μ* =100 *mm* is the x-coordinate of the maximum pain point. The deviation of the last palpation coordinate from *μ* indicates the localisation error for the trial.

Figure 4 shows an example of palpation force and the x-position against time of one naive participant for one trial with facial and vocal pain expression feedback. When the participant palpates the abdomen, the force sensor platform measures the continuous force and the x-position. To discretise the data, the force of a palpation point is defined by the local maximum force indicated by the orange dot at the peak in Figure 4. The recorded values of palpation force are comparable with previous studies on the topic (Konstantinova et al., 2014). Once the peak of palpation force is found, the time stamp of the palpation point is recorded. The x-position of the palpation point is extrapolated from the time stamp which is also shown with an orange circle. *μ* indicates the position of the maximum pain point. The difference between the x-position of the palpation point is shown with an orange circle, and the line *μ* is the distance from the current palpation point to the maximum pain point. Participants palpated the abdomen approximately 14 times for this trial before determining the maximum pain point. This is seen from 14 peaks in both the plot of palpation force against time and the x-position against time. The palpation force was relatively constant in this trial.

**FIGURE 5:**
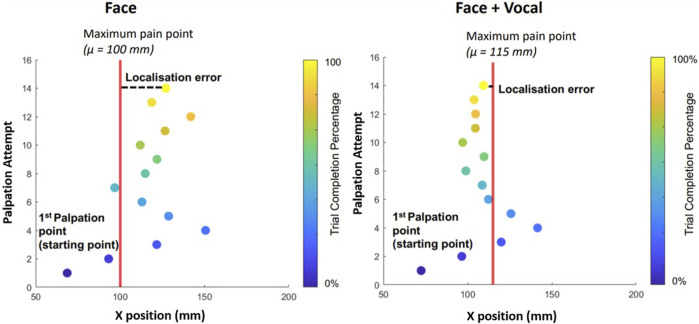
On the left is the palpation trajectory from a trial with only facial expression feedback. On the right is the palpation trajectory from a trial with both facial expression and audio pain expression feedback. *y* − *axis* shows the palpation attempt. *x* − *axis* represents the x-position of the respective palpation attempt. For example, during the trial involving only facial expressions, the participant was able to determine the maximum pain point after 14 palpation attempts. Each filled scatter circle represents a palpation attempt, and the vertical line represents the x-position of the maximum pain point in the respective trial. Palpation points are colour coded according to the trial completion percentage (see the colour bar on the right).

Figure 5 shows an example palpation trajectory by one of the naive participants with and without vocal pain expressions. The red vertical line shows the location of the maximum pain point *μ*. The distance from the x-position of the palpation attempt to the line is the error of each palpation attempt from the maximum pain point. Each scatter point shows a palpation attempt and its x-position on the abdominal phantom. Each point is colour coded according to the trial completion percentage where lighter colour of the point indicates the trial completion percentage approaching 100%. The participant starts the trial on the right of the silicon phantom, around 70 *mm* as instructed. The distance of the final palpation point to *μ* is the localisation error. From Figure 5 (left) and Figure 5 (right), it can be seen that the participant starts palpating from the right along the x-direction but reverses the direction once the facial and vocal pain expression shows less pain. There seems to be an oscillation around the maximum pain point suggesting the participant is attempting to determine the exact maximum pain point. For this particular trial from this particular participant, the oscillation for the trial with facial and vocal pain expression feedback has lower amplitude of oscillation around the *μ* and achieved smaller localisation error at the last palpation attempt.

**FIGURE 6:**
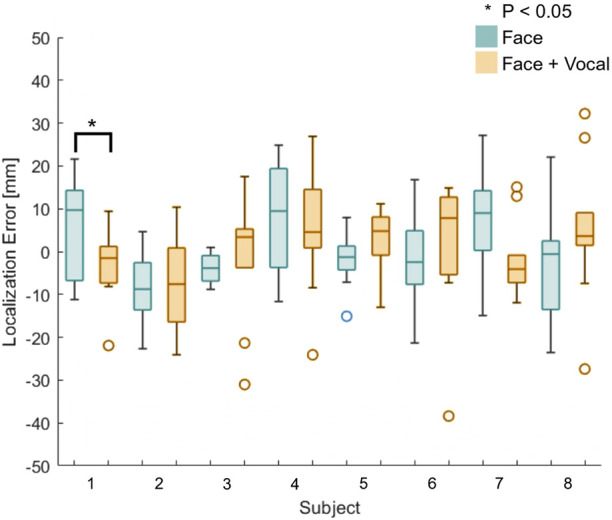
Box plots of the localisation error between the identified and actual maximum pain point for all naive participants (*n* = 8). Participant 1 shows statistically significantly reduction in the localisation error when he/she was presented with both the vocal and facial pain expressions.

Figure 6 shows box plots of the localisation errors of all eight naive participants. Blue plots represent trials without vocal pain expression feedback, and orange plots represent trials with both facial and vocal pain expression feedback. The results from this experiment came from a normally distributed population according to the Anderson–Darling test. The localisation error between the two approaches was not statistically significantly different, according to a two-sample t-test applied to the localisation errors between approaches with and without vocal pain expression feedback. Then, a two-sample t-test was applied to the localisation error between two approaches of each subject. Only participant 1 showed a statistically significant reduction in the localisation error (*p* < 0.05) when he/she performed with both types of feedback. This is shown using a star symbol above the box plot for participant 1. For participants 2–8, there was no statistically significant difference in localisation error between the two approaches.

**FIGURE 7:**
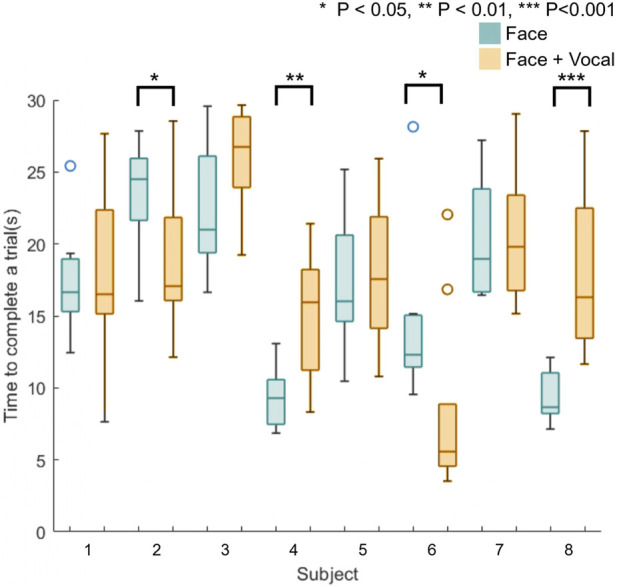
Box plots of time taken to find the maximum pain point for all naive participants (*n* = 8). Four participants (S2, S4, S6, and S8) show a statistically significantly difference in the time to complete a trial between two approaches.

Figure 7 shows box plots of the time taken to complete the trials for all eight naive participants. Blue plots represent the time taken to complete a trial without vocal pain expression feedback, and orange plots represent the time taken to complete a trial with both facial expression feedback and vocal pain expression feedback. Using the Anderson–Darling test, the results from this experiment were not from a population with normal distribution, and therefore, the Mann–Whitney U test was used to calculate the statistical significance between the time taken to find the maximum pain point between the two approaches. The result from the Mann–Whitney U test applied to the time results of the first and second group of participants suggests that there is no statistically significant difference between the approach without vocal pain feedback and the approach with vocal pain feedback.

Applying the Anderson–Darling test to the data from individual participants showed that the time data do not form a population with normal distribution. The Mann–Whitney U test was applied to the time data from each subject. Half of the participants showed a statistically significant different time taken to find the maximum pain point between two approaches. This is shown using star symbols above the box plot for participants 2, 4, 6, and 8 in Figure 7, whereby a higher number of stars indicates larger *p*-value. These participants started the first 10 trials with only facial expression feedback and completed the next 10 trials with facial and vocal pain expression feedback. Although two out of four of the participants (subject 2 and 6) took statistically significantly less time to complete a trial with the face and audio feedback approach, two out of four of the participants (subject 4 and 8) took statistically significantly more time to complete a trial with the face and audio feedback approach.

**FIGURE 8:**
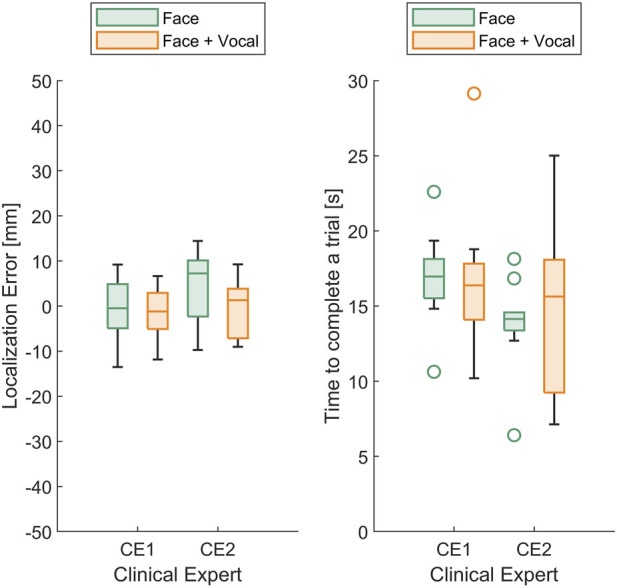
(Left) Box plots of the localisation error between the identified and actual maximum pain point and (right) box plots of time taken to find the maximum pain point for the two (*n* =2) clinical experts.

In Figure 8 (left), the box plots illustrate the localisation error between the identified and actual maximum pain points for the two clinical experts. Although the median localisation error of CE2 during trials where the participant presented both facial and vocal pain expressions is smaller compared to trials with facial expressions alone, no statistically significant difference between the approaches was found. In Figure 8 (right), the box plots display the time taken to find the maximum pain point for the two clinical experts. The results indicated that there is no statistically significant difference in maximum pain point estimation time between the two approaches.

Table 1 summarises the 5-point Likert scale questionnaire answers of the two clinical experts. Both participants strongly agreed that vocal pain expressions were helpful in identifying the maximum pain point. Three vocal pain expressions were highly distinguishable for both participants, while they scored differently but, on average, leaned towards agreement regarding the realistic nature of the vocal pain sounds synthesised during the experiments. On average, both participants agreed that the proposed simulator platform with vocal pain expressions is useful or potentially useful in palpation medical training.

**TABLE 1:** 5-point Likert scale questionnaire answers (where 1 = strongly disagree and 5 = strongly agree) by the two clinical experts.

Question	CE1	CE2
(1) Were the vocal pain expressions realistic?	4	3
(2) Were the vocal pain expressions clearly distinguishable?	5	4
(3) Were the vocal pain expressions helpful in identifying the maximum pain point?	5	5
(4) Is the simulator platform with vocal pain expressions useful or potentially useful in palpation medical training?	4	3

For the open-ended question regarding improvements needed in terms of vocal pain expressions, one participant noted that the robopatient should be able to simulate patients with different pain sensitivities during training. The other participant highlighted the issue of simulating patients who rarely produce vocal pain expressions. Instead of clear vocal pain expressions, these patients tend to produce grunts. The participant emphasised the importance of simulating such cases. For conditions like appendicitis, it is crucial to identify subtle vocal pain expressions alongside facial expressions. For the second open-ended question on what features would you like to see in a medical simulator with vocal pain expressions, one participant suggested including diverse vocal pain expressions to represent a wide range of patients. The second participant suggested expanding the robotic patient platform to include more systematic and clinically realistic palpation simulations, encompassing scenarios of both superficial and deep palpation, as well as instances of rebound pain in patients.

## 4 Discussion

In this paper, we presented a robopatient with vocal pain expressions for abdominal palpation training. The proposed system is capable of rendering real-time pain facial expressions and vocal pain expressions given the user palpation force and position on a silicone abdominal phantom. We proposed a threshold-based algorithm for generating the vocal pain expressions based on the estimated pain intensity of the robopatient. The results from the pilot study that involved naive participants (*n* = 8) showed that despite no significant differences in localisation error and estimation time between facial pain expression-only and facial with vocal pain expressions feedback as a group, data from one participant showed a significant reduction in localisation error when both types of feedback are presented. Half of the participants showed a significant difference in estimation time between two feedback approaches.

Qualitative post-experiment feedback indicated that when vocal pain expression is present, participants start to focus more on the vocal expressions due to the discrete nature of the cues which could be the reason for the non-statistically significantly difference in the localisation error between two approaches since there is a range of maximum pain intensity for the maximum vocal pain expression. Nevertheless, the majority of the participants reported that the facial with vocal pain expressions feedback approach made it easier for them to make the decision. The differences in the localisation error between the two approaches could be significant if participants had more time to familiarise themselves with the system to reach the peak of their learning curve. Moreover, in our pilot study, we tested the system with two clinical experts (*n* = 2). Their qualitative post-experiment feedback on the system indicated that vocal pain expressions are a valuable and essential feature in palpation simulators. They also recommended enhancing the proposed robopatient simulator by incorporating diverse vocal pain expressions, such as pain sounds like grunting, and by introducing more comprehensive and clinically oriented palpation training programs. These programs would encompass scenarios involving superficial and deep palpation, as well as training for identifying rebound tenderness.

Despite these positive findings, there are several limitations in this study. There were only three levels of the pain to sound map; there is a range of maximum pain intensity for the maximum vocal pain expression. Hence, participants may find it difficult to distinguish the feedback associated with the maximum pain when they palpate near the goal. One limitation of our study is that we had a relatively small sample size of naive participants. This may have reduced the statistical power of our analysis and led to statistically non-significant outcomes. We presented this study as a pilot investigation designed to demonstrate our approach of integrating pain vocal feedback to robopatients for abdominal palpation training. Moreover, we did not consider effects of the gender, ethnicity, and prior palpation experience of the participants in the experiment. This limited the examination of whether and how participant demographics and background (e.g., medical students who already had palpation training) influence performance when interacting with the robopatient with the vocal pain expressions. We plan to expand our investigations with a larger number of participants from diverse genders and ethnic backgrounds and participants with different palpation experience levels to address these limitations. Another limitation of our study is the use of male pain sounds and a white male face on the developed robopatient, which may limit the generalisability of our findings to other gender and ethnic groups. We acknowledge the need for future research to investigate the impact of gender and ethnicity on pain perception in terms of facial and vocal pain expressions during abdominal palpation. Therefore, our future plan will include the use of facial and vocal pain expressions from different ethnic and gender backgrounds on the robopatient to investigate potential cross-cultural differences in pain perception. In actual palpation by physicians on patients, there is no time limit. However, in this study, we introduced a time constraint on users to complete the task. This constraint may have influenced participants’ performance as they were under pressure to complete the trial within the given time. We have not investigated this aspect in our current work but plan to explore the effects of time constraints on user performance in future research. In this pilot study, we evaluated our system with only two clinical experts. However, we acknowledge that involving a sufficient number of expert clinicians and/or senior medical students would be crucial in assessing the simulator’s effectiveness in training novice medical professionals. Conducting future studies with a larger cohort of clinicians and/or medical students to evaluate the simulator’s efficacy remains an important direction for our further research.

Nevertheless, we have developed a controllable and customisable physical robopatient with facial and vocal pain expressions for abdominal palpation training. The developed robotic patient is controllable in terms of facial expressions, pain vocal expressions, and mapping functions between palpation and vocal and facial expressions. This controllability allows us to simulate different pathological conditions of patients during palpation training and explore different mapping models between palpation behaviour and vocal and/or facial expressions. The customised system can be tested on a diverse participant pool to improve the understanding of human reactions to pain, establishing new opportunities for future research on vocal pain expressions during abdominal palpation. Future directions of this paper are based on improving the robopatient, such as adding bowel sounds, which is common during abdominal examinations. We envision that our study provides a foundation for future research in this area.

## Data Availability

The original contributions presented in the study are included in the article, further inquiries can be directed to the corresponding author.
